# Communicating Arsenic’s Risks

**DOI:** 10.3390/ijerph16183436

**Published:** 2019-09-16

**Authors:** Shannon H. Rogers, Laurie R. Rardin, Kathrin Lawlor, Celia Y. Chen, Mark. E. Borsuk

**Affiliations:** 1 Cooperative Extension, University of New Hampshire, Durham, NH 03824, USA; 2 Dartmouth Toxic Metals Superfund Research Program, Dartmouth College, Hanover, NH 03755, USA; laurie.r.rardin@dartmouth.edu (L.R.R.); kathrin.lawlor@gmail.com (K.L.); celia.y.chen@dartmouth.edu (C.Y.C.); mark.borsuk@duke.edu (M.E.B.); 3 Present Address: Department of Civil and Environmental Engineering, Duke University, Durham, NC 27708, USA

**Keywords:** mental models, arsenic, environmental communication, risk communication, community based research

## Abstract

Arsenic is a naturally occurring toxic metalloid that has many human health implications. Its strong prevalence in the bedrock and thus much of the well water in New England puts many private well owners at risk. It is also found in food products, particularly those that contain rice. Despite the documented health risks, arsenic is not high on the list of concerns for residents of the region. This study will describe two types of environmental communication efforts that have been undertaken by the Dartmouth Toxic Metals Superfund Research Program (DTMSRP)—the development and evaluation of a comprehensive website, Arsenic and You, and a mental models research approach to better understand the disconnect between expert and community perceptions of arsenic risk. We find that there are knowledge gaps between the two, particularly regarding the origin of arsenic in drinking water and food, the necessity of testing well water, and the process for treating water that is above recommended limits. Moreover, the mental models approach provides a structured framework for better understanding these gaps. A website can address some of these disconnects, and it is important to have a “one-stop shop” for vetted information on the risks and steps to reduce exposure.

## 1. Introduction

Inorganic arsenic (As), although naturally occurring, is classified by the International Agency for Research on Cancer as a group 1 carcinogen [1]. Across the globe 140 million individuals are exposed to toxic levels of As in drinking water [2]. In the United States, approximately 2 million individuals drink private well water that exceeds the Environmental Protection Agency (EPA) limit for As of 10 parts per billion (ppb). In New Hampshire, approximately 61,000 drink private well water containing greater than 10 ppb of As [3]. Arsenic is a naturally occurring metalloid found in the earth’s crust. Arsenic is also in the air, water, and soil. Contamination from arsenic can be naturally occurring, or may come from anthropogenic sources. By far the most widespread arsenic exposures have been from natural sources. Exposure through water and food are the most common routes of exposure. Other paths of exposure include smoking, use of legacy pressure-treated wood products (pre-2003), occupational exposures, and proximity to an arsenic polluted site. Because arsenic is tasteless, colorless, and odorless, and because most exposure in the US does not cause immediate adverse health effects, many people are not aware of their arsenic exposure. 

A major goal of the Dartmouth Toxic Metals Superfund Research Program (DTMSRP) is to increase awareness of adverse health risks related to arsenic and to provide solutions to reduce arsenic exposure in water and food. A recently published emerging issues brief through the New Hampshire Comprehensive Cancer Collaborative [4] summarizes the risks and health impacts of arsenic in water and food. New Hampshire has some of the nation’s highest rates of the types of cancer that can be related to arsenic exposure. Despite these conditions, citizens lack an awareness of the risks of drinking water with high levels of As and eating food with As in it. Part of the reason for this lack of awareness comes from the challenge of communicating risks about complicated toxic exposure routes. Communication is often done ad hoc, without empirical analysis, because of resource and time constraints.

The solution to ineffective, ad hoc risk communication is structured engagement with target communities to collaboratively reveal what people already know relative to what they need to know to make informed decisions. Subsequent communication (broadly defined here to include verbal exchange, informational materials, training programs, educational curricula, and risk assessment tools) can then be focused on filling these gaps in knowledge, so long as such knowledge is available from any of the (academic or non-academic) partners. If not, then relevant new research projects— ideally in collaboration with the communities themselves—can be initiated. This can be described as “actionable two-way communication”. The method that we have used to structure community engagement is the mental models approach. The term “mental model” was used in this capacity by Bostrom et al. [4] to describe the set of concepts people use to understand the causes, consequences, and control of a particular risk. These beliefs may be highly detailed and quantitative, or they may be ill-formed and fragmentary. The important point is that a mental model is an allegory for what a person currently believes regarding a potential hazard. In this paper we will discuss how we have used the mental models approach to understand the difference between expert and community perceptions of risk from exposure to As. These insights go hand in hand with efforts to provide a “one-stop” source for beginning to understand the risks of exposure to As through water and food with the Arsenic and You website https://www.dartmouth.edu/~arsenicandyou/.

We discuss two types of environmental communication efforts that have been undertaken by the Dartmouth Toxic Metals Superfund Research Program—the development and evaluation of a comprehensive website, Arsenic and You, and a mental models research approach to better understand the disconnect between expert and community perceptions of arsenic risk. We found that there are knowledge gaps between the two, particularly regarding the origin of arsenic in drinking water and food, the necessity of testing well water, and the process for treating water that is above recommended limits. Moreover, the mental models approach provides a structured framework for better understanding these gaps. A website can address some of these disconnects, and it is important to have a “one-stop shop” for vetted information on the risks and steps to reduce exposure.

## 2. Materials and Methods

**Figure 1:**
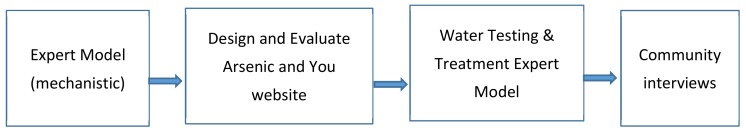
Steps in the iterative process of arsenic (As) risk communication research.

We describe an iterative process of research and communication that was combined to learn about the most important components of understanding perceptions of arsenic exposure through food and water. It was also designed to better understand the barriers to communication by comparing “expert models” of how a system works with community-level understanding. Figure 1 shows the flow of the steps in our risk communication research process, and will be referenced throughout the paper. Essentially, we used this four-step process to collect information on expert knowledge, then used these insights to inform a website for public health and environmental risk communication. Simultaneously, we have also been trying to understand layperson perceptions of risk to then compare with expert understanding and better inform future communication efforts.

### 2.1. Creating the Expert Model (Mechanistic)

The mental model process has two parts: (1) interviews of scientists and environmental and health agency officials to establish an expert model of the relevant risks, and (2) a series of interviews of organizational partners and community members to assess popular beliefs and their correspondence with the expert model. The mental models research in our work was interwoven with the development and evaluation of a website.

The purpose of establishing an expert model is to explicitly capture the current state of knowledge regarding the causes, consequences, and points of control of a particular risk. The goal is to create a single-pooled description of the combined understanding of the scientific community, not simply the beliefs of single expert. The process involves iterative exchange with experts to review and clarify the model representation, which forces the experts to think systematically about their beliefs and the connections with those of other experts. Morgan et al. [5] have provided a detailed description of the approach as well as practical lessons learned. We followed this process very closely.

**Figure 2:**
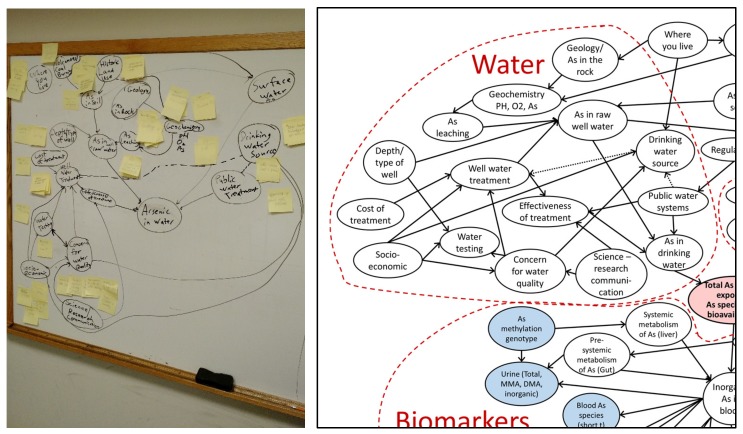
Brainstorming and sticky notes become a detailed mental model. The representation on the **right** is not meant to be read node by node, but rather to show the type of graphical model that results from the assembly process (**left**).

In 2016, a group of approximately 10 experts affiliated or collaborating with the Dartmouth Toxic Metals Superfund Research Program gathered together to create the first expert mental model. The group included epidemiologists, biologists, environmental communication experts, microbiologists and immunologists, and an engineer. We utilized the assembly method of creating the expert model. In this method, experts were gathered together and asked to write down all of the factors that impact a person’s exposure to arsenic. These factors were written on post-it notes that were grouped into categories based on group suggestions, with each category becoming a node (e.g., historic land use). Then, experts worked together to connect nodes using arrows to indicate influence. Figure 2 shows how brainstorming and sticky notes become a mental model of how arsenic risks are generated.

**Figure 3:**
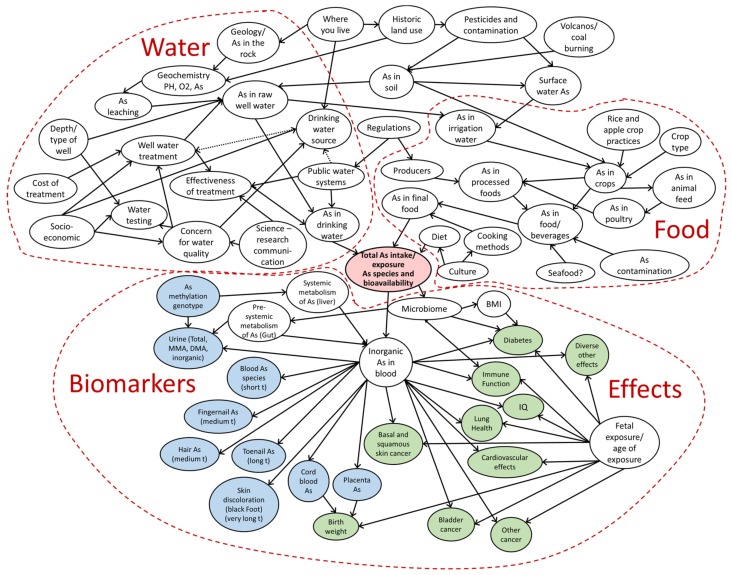
Dartmouth Superfund Research Program (DTMSRP) team expert model.

The full expert model that developed out of this intensive process, lasting several hours, is displayed in Figure 3. Because the model is very complex it is challenging to display it in a way where all of the nodes are legible, so Figure 3 is meant to represent a visual of the scope of the model and Table 1 breaks down all of the nodes in Figure 3. (A more detailed version of Figure 3 is available in the Supplementary materials). The group organized the nodes into four main areas: water, food, biomarkers, and effects. This model represented a comprehensive look at the mechanistic components of arsenic exposure risk. It was heavily focused on some of the medical and epidemiologic components of risk.

The experts in this process went into depth when asked about the factors that impact arsenic exposure risk. There was a rich discussion about the mechanistic components of exposure with a deep dive into biomarkers, including emerging work on the microbiome. All of the nodes detailed in Figure 3 are included in Table 1.

### 2.2. Design and Evaluation Arsenic and You Website

Many adults in the US use websites on the internet to obtain information on public health issues such as exposure and effects of environmental toxicants. According to the Pew Research Center, 89% of American adults use the internet, 72% of those users looked up online health information, and 77% of adults own smartphones [6]. This makes digital information on public health a good avenue for connecting with many types of audiences. While there is this opportunity, there are also some challenges to communicating online, including the various knowledge gaps audiences might have, providing actionable information when science is still developing, and defining terminology [7].

All this being said, websites are often used as risk-communication tools. We had several goals with the website, including the following:

1. to establish a centralized resource for information on sources of exposure and the effects of arsenic;
2. to establish a comprehensive information source on arsenic in food, water, and from other sources;
3. to establish a tool to help visitors make informed decisions in reducing their arsenic exposure; and
4. to establish a clearinghouse of information linking resources from a range of institutions.

With these goals in mind and informed by the creation of the expert mental model—particularly the strong emphasis on water and food as routes of exposure (with food being a relatively new and emerging field of exposure (e.g., Lai et al. [8])—we took the following steps to create the website:

- reviewing the expert model described above;
- creating a project advisory team to represent diverse communities relevant to arsenic exposure and health risks;
- consulting scientific experts (many of whom were involved in the creation of the expert model discussed above) during content development and review;
- using focus groups and user testing to obtain feedback on uncertain science;
- using best practices for online public health communication;
- developing a text based on health literacy and numeracy best practices;
- creating a disclaimer regarding the extent of scientific knowledge and recommended actions; and
- hiring a public health consultant to create fact-based images to convey complex information and evaluate the website.

With a national focus, the website was designed to provide information to the public with a specific focus on those who: have small children; drink water from a private well; eat diets high in rice or rice-based products, including those following a gluten-free diet; live near hazardous or industrial waste sites; smoke cigarettes; or may come into contact with pressure-treated wood. The expert model identified these characteristics as the most-related to increased potential for exposure to arsenic.

With arsenic in particular, the complexity of the element and exposure risks presents challenges in lowering exposure, and risks and benefits of recommendations for dietary exposure have not been assessed. As such, we have not yet been able to provide the measured quantities of arsenic in food or beverages users want to help them make choices on consumer purchases.

As a Superfund Research Program Center focusing our research on human exposure to arsenic from drinking water and food, we developed the Arsenic and You website (https://www.dartmouth.edu/~arsenicandyou/) to provide comprehensive information on arsenic in food, water, and from other sources for a primary audience of families, caregivers, and the general public. The Arsenic and You website also responds to needs identified by members of the New Hampshire Arsenic Consortium for a central, web-based clearinghouse of information on arsenic.

Through the development of this site we have learned that clear and actionable communication is challenging when scientific understanding of an issue is still developing. In many areas, arsenic science is far from conclusive. For example: the risks and benefits of recommendations around dietary arsenic have not been directly assessed; messages vary widely between organizations and over time as arsenic science moves forward; and, many important knowledge gaps exist.

In order to address these challenges, we developed a website design process that: involved arsenic experts and public health communicators from the outset; incorporated audience feedback at multiple stages of design; utilized our Superfund Research Program (SRP) network for national audience testing and to vet, review, and sign-off on each web page; and included statements to site visitors clarifying the limits of the information provided. In the end, the engagement of both arsenic experts and the intended audience helped us to identify gaps in the general public’s understanding of arsenic, and allowed us to determine the most effective ways to communicate both the science and best practices for protecting public health.

### 2.3. Refining the Expert Mental Model by Adding a Water Testing and Treatment Perspective

After the creation of the initial expert model and the development of the website, we conducted another workshop with state regulatory experts that focused largely on the exposure factors related to water. Because of our close partnership with the State of New Hampshire and our mandate to serve the region—a region that has high levels of arsenic in well water and elevated levels of bladder cancer [9]—we wanted to further build on the drinking water exposure area of our understanding.

**Figure 4:**
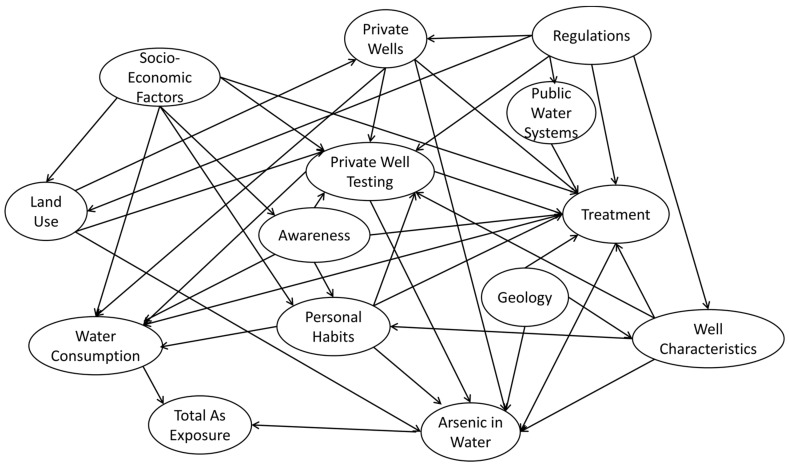
Additional expert model focused on exposure risk from drinking water.

In late 2016, we gathered a group of experts from state health and environmental offices and prompted them with the same question: “What are the factors that affect an individual’s exposure to arsenic.” This time we were specific and added “exposure from drinking water” to the prompt. The discussion from this group of experts focused heavily on the factors that impact well-water testing, treatment, and water consumption (as demonstrated by the number of linkages made to those nodes—7, 10, and 6, respectively). This model, highlighted in Figure 4, is less complicated and easier to read than the initial expert model in Figure 3 as it was focused solely on As exposure via water.

### 2.4. Community Interviews

Before detailing the next step in our process, the community-level interviews, we will provide context for selecting the communities of focus. One of the major community focus areas in the Community Engagement Core of the DTMSRP consists of households that obtain their drinking water from private wells potentially contaminated with arsenic. Since under current EPA regulations there is no federal agency mandated with ensuring the safety of privately owned wells, we believe that town officials represent a promising partner to support household well testing and treatment. Therefore, we worked with two towns in southeastern New Hampshire that are known to be at risk. These towns, Raymond and Derry, have: (i) a majority of residents who drink from private wells; (ii) a high percentage of well-water samples tested by the NH Department of Environmental Services (DES) that exceed the maximum acceptable arsenic level set by the EPA; (iii) Superfund sites at which present or past land-use practices might account for contaminants in ground water; (iv) proximity to geologic units likely to contain naturally occurring arsenic; and (v) limited resources or means for notifying well owners about the risks of arsenic in their water.

Raymond, NH, is located in the western part of Rockingham County (Figure 3), and has a population of approximately 10,399, which ranks it 28th among NH’s incorporated cities and towns; the population density is approximately 380 people per square mile. A Superfund site, the former Mottolo Pig Farm, is located approximately three miles south of Raymond’s center. In the late 1970s, the owner of the property disposed of chemical manufacturing wastes from two companies into a 1/4-acre fill area. Subsequent studies showed that groundwater beneath the site was contaminated and that these contaminants were seeping into a brook on the property that eventually empties into the Exeter River. The Exeter River is a source of drinking water for the nearby communities of Exeter, Hampton, and Stratham. An additional 1600 people within three miles of the site depend on ground water as a source of drinking water. State officials remain concerned about public health issues in Raymond because in 2009 NH DES sampling results revealed that many of the nearby residential wells had elevated concentrations of arsenic. The former disposal area is located on an undeveloped wooded lot, but there are approximately 200 single family residences located within one mile of the site, with the nearest residence located approximately 600 feet to the west.

Derry, NH, also in Rockingham County, has a population of 33,109, which makes it the fourth most populous community in New Hampshire. Three Superfund sites surround the town of Derry: Auburn Road Landfill, Tinkham Garage, and Town Garage/Radio Beacon. Auburn Road was an active landfill until the state ordered it closed in 1980 after identifying hazardous wastes in soil, surface water, and ground water. In 1986, the EPA determined that contaminated ground water was flowing off-site toward drinking water supply wells at a mobile home park and potentially towards other residential wells in the area. Approximately 1000 people live within three miles of the site. Two unnamed streams drain from the site and eventually empty into the Merrimack River. An EPA study is being planned to assess arsenic transport processes and potential geochemical reactions controlling arsenic concentrations at the landfill site. 

**Figure 5:**
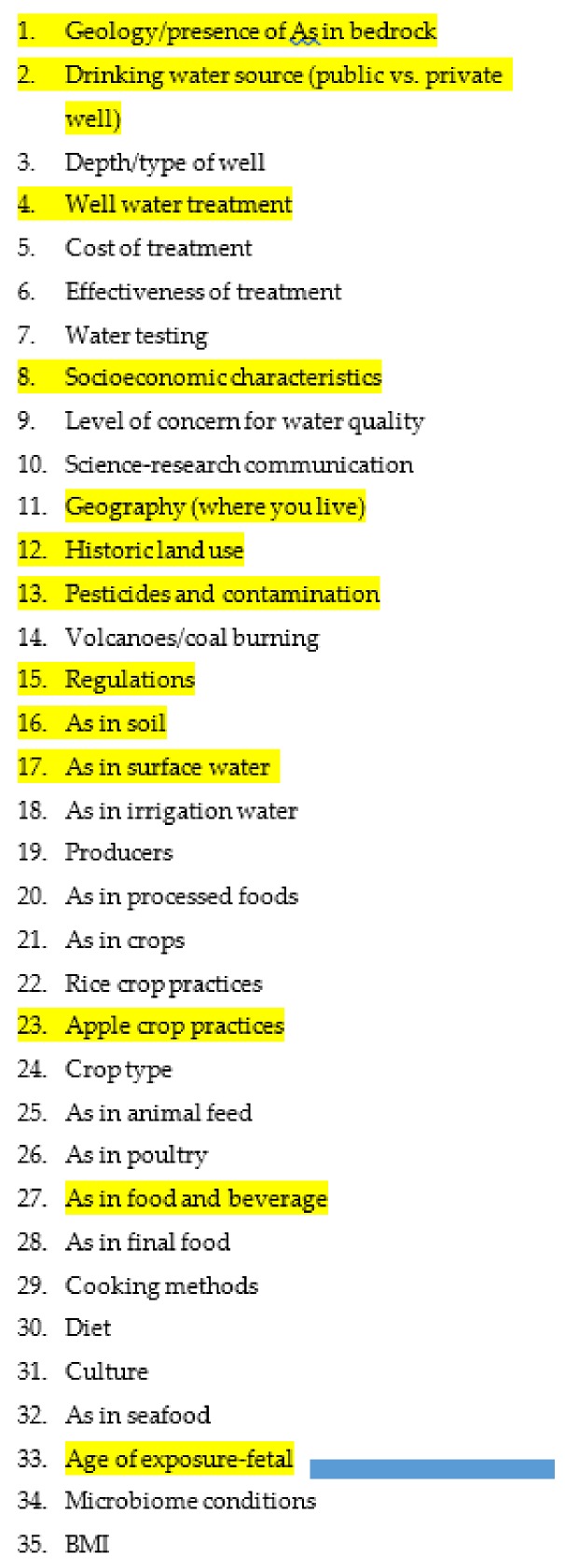
Nodes used to develop community interviews. Highlighted nodes indicate themes that were discussed in both the expert models and the community interviews.

Now that we have provided background on the communities we focused on for the interview portion of the research process, we can explain that process in detail. The next step in the mental model process is to combine any similar nodes from the expert model. We did this for the most prominent nodes from each of these models and developed a list of 35 nodes of importance to include in the community-level interviews. Those nodes are listed in Figure 5 and were used to create an interview protocol that was administered to 21 community members from our two focus communities: Raymond, NH, and Derry, NH, in the Fall of 2017. Our research with human subjects was approved by the Dartmouth Internal Review Board and participants were recruited via newspaper and Facebook ads. They were provided with information on the study, consent forms, and an option to stop the interview at any time. We conducted the interviews in community locations, including the town hall and a community health center. Participants were provided with a $50 gift card for their time and travel.

**Figure 6:**
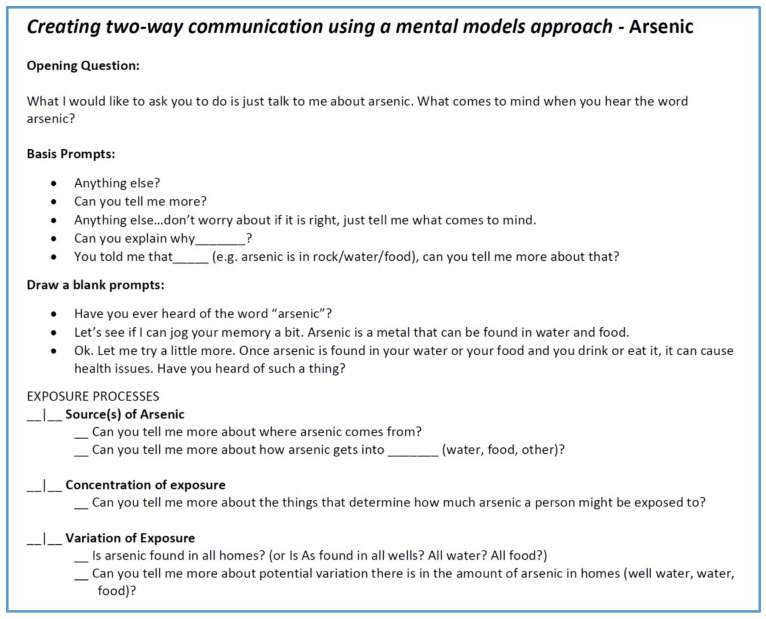
Community interview protocol.

A mental model interview is meant to start very broadly and then narrow into a more specific discussion of the potential risk or issue. Figure 6 is a portion of our interview protocol. We began with the statement “Tell me what comes to mind when you hear the word arsenic”. The interview protocol was then organized into several different sections we called “exposure processes”, “effect processes”, “risk assessment and management”, and “risk comparisons”. The full interview protocol is available in the Supplementary Materials.

Interviews ranged from 20 min to an hour and were voice recorded and then transcribed verbatim by two of the authors. The interview transcripts were then reviewed for the nodes from the expert model and for any other emerging “nodes” or themes. Themes that were most frequently mentioned in the interviews are highlighted in Figure 5.

## 3. Results

### 3.1. Website Statistics

**Figure 7:**
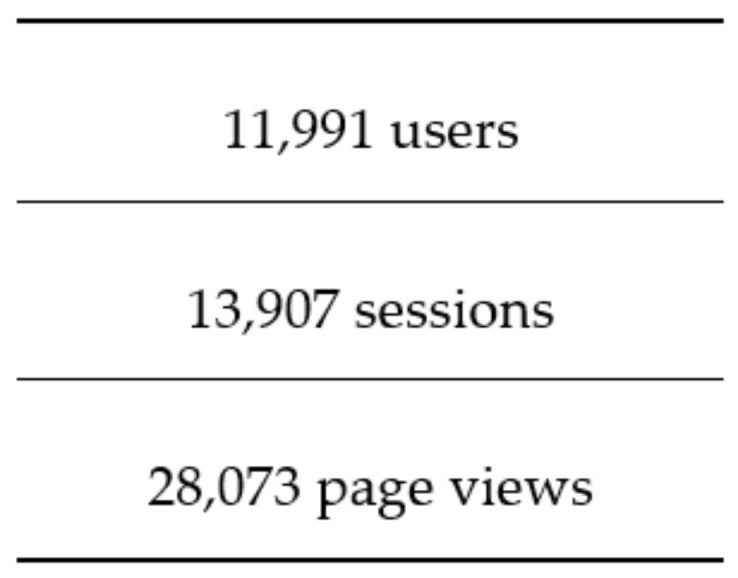
Summary of Arsenic and You website analytics. Data comes from Maccini 2018 [10].

Promotion and evaluation of the website was conducted from February 2017 to May 2018. During this period, the website Arsenic and You was promoted at 34 in-person events, via 4400 individual emails in nine campaigns, and through 14 Facebook posts that reached 3200 Facebook users. Ten partners added Arsenic and You to their own websites as a referral link and 100 organizations were contacted to build partnerships. This led to website analytics described in Figure 7 with sessions originating from every state in the country.

#### Website Evaluation Findings

Findings from a brief evaluation survey on the website indicated that 86% of survey respondents (*n* = 32) reported learning something from the website in one or more key areas. One-hundred percent (100%) of evaluation survey respondents reported that they had made a change or plan to make a change related to exposure to arsenic as a result of what they read on the Arsenic and You website.

### 3.2. Findings from Community Interviews

One of the best ways to highlight findings from qualitative analysis of interview text is to share themes and representative quotes. Of the 35 nodes from the expert model, 15 nodes were mentioned by more than one interviewee. Additionally, two new nodes were mentioned by more than one interviewee—issues that related to the real estate transaction process (“real estate”), and discussions related to a neighbor’s or friend’s knowledge of As exposure (“neighbor or friend”). Table 2 highlights example quotes from these themes.

## 4. Discussion

The risk perception research by DTMSRP has a goal of informing and improving public health communication. The detailed, empirical process of risk perception research described here is fairly unique for a low-dose, long-term exposure risk like arsenic. The methods and results of this process are interwoven and connected—the creation of an expert model of the factors that contribute to arsenic exposure risk established a method, but also provided important results that were used to create the first website for one-stop information on the risks of arsenic from food and water. The expert model process highlighted the emerging importance of providing information on the risk of exposure from both water and food.

While not designed as a test, the community interviews do highlight some of the inaccuracies in everyday understanding of how the processes work. The difference between the 35 nodes of the expert model and the 15 nodes mentioned in the community interviews also demonstrates the differences in the complexity of understanding between experts and community members. This makes sense because of the training and focus of experts, and we cannot expect community members to be well-versed in all of the minutia of complicated chemical and biological processes that lead to harmful effects from arsenic exposure [11]. However, it can be important for experts, particularly those who work in communicating to public audiences, to see those differences. In our specific study we see that the public does not have a strong understanding of the biological processes involved in how the body reacts to arsenic, nor do they have a strong awareness of the need to test and then treat drinking water that may contain elevated levels of arsenic. We do know that there are challenges in communicating the risks of natural versus manmade risks/disasters [10], and part of this may explain the lack of urgency and interest in arsenic exposure. Most of our interviewees knew that arsenic was naturally occurring. Other manmade chemicals in drinking water were high on the minds of residents of our communities [12]. Importantly, our community interviewees highlighted two nodes that no experts had mentioned, the importance of the real estate transaction process for communicating water quality risks, especially from well water, and the importance of friends’ and neighbors’ experiences in sharing information on risks to homeowners. These two areas are prime for future communication collaboration opportunities, particularly the home-purchasing process.

Many of our community interviews began with interviewees indicating that poison was the first thing they thought of when they heard the word arsenic, often due to reading a crime book or watching a movie. However, by the end of the interview, many felt that arsenic was a low risk compared to other issues in their lives. This shows a strong disconnect that we are planning to further investigate with a broad scale survey of the public in the southeastern part of the state. This process will help us confirm or refute the findings from the community interviews.

With all research involving human subjects there are caveats to discuss, and we found that many of our interviewees had done some research prior to meeting with us, even though the recruitment information indicated that they should not research the topic. A group of 20 community interviews can be a sufficient sample for making generalizations about a community, especially when theme saturation is observed [13]. However, conducting a broader survey can help us to confirm and compare the findings from the smaller N, and we look forward to that next step in the process. It will also inform updates to the Arsenic and You website and future risk communication work by our Superfund Research Program and partners.

## 5. Conclusions

In this paper we shared two connected approaches to understanding risk perceptions and communicating risks, as well as how they can inform one another. Differences between experts and community knowledge, and perceptions of arsenic risk were apparent, and can be used to inform further communication. By highlighting areas of concern or misunderstandings by community members, experts can better focus their research and communication on clarifying those disconnects. Expert models tend to be far more detailed and mechanistic than community understanding of this risk. We found that there are knowledge gaps between experts and community members, particularly regarding the origin of arsenic in drinking water and food, the necessity of testing well water, and the process for treating water that is above recommended limits. However, community members also have important insights—in this case, they highlighted a new opportunity to collaborate with realtors and others involved in the home-purchasing process to communicate the risks of arsenic in well water. Community insights have also motivated discussion about including information for home buyers and renters regarding water quality testing at the time of this important decision/transaction. This will be a fruitful avenue for future research, but also one that is not without its challenges.

The Arsenic and You website has reached a broad audience and is providing actionable information. Prior to this work, there was very little information for consumers on the risks of exposure from food and actionable steps that could be taken to reduce exposure. The website has been informed by the mental model research process to date. Future iterations and updates will continue to be informed by the findings from the community interviews and confirmatory survey.

Moreover, the mental model approach provides a structured framework for better understanding gaps in knowledge and opportunities for public health communication about risks related to any number of environmental issues. A website can address some of these disconnects, and it is important to have a “one-stop shop” for vetted information on the risks and steps to reduce exposure.

## Figures and Tables

**Table 1:** Categories and specific nodes from the Dartmouth Toxic Metals Superfund Research Program (DTMSRP) team expert model.

Water.	Food	Biomarkers	Effects	Other
Geology	As in irrigation water	As methylation genotype	Diabetes	Where you live
Geochemistry	Producers	Urine	Immune function	Historic land use
Drinking water source	As in processed food	Blood As species	Intelligence Quotient	Pesticides and contamination
Public Water Systems	Seafood	Fingernail As	Lung Health	Volcanoes/Coal Burning
Depth/type of well	Cooking methods	Hair As	Cardiovascular effects	Regulations
Well water treatment	Diet	Toenail As	Bladder cancer	As in soil
Effectiveness of treatment	Culture	Skin discoloration	Basel and squamous skin cancer	Surface water As
Public water systems	Rice and apple crop practices	Cord blood As	Other cancer	Metabolism
Socioeconomic factors	As in Animal feed	Placenta As	Diverse other effects	Microbiome
				Body Mass Index
				Science-research communication

**Table 2:** Themes and quotes from community interviews.

Theme/Node	Representative Quote
Geology/presence of As in Bedrock	*Because it is naturally occurring, I want to think it is something that can already be in the ground and contaminate water sources. I want to think that is one of the reasons you are allowed to have some much of it in your system without it being poisonous.*
Drinking water source (public vs private well)	*I don’t really know that much about it, it’s just that I know it’s a potential hazard if you have private wells. Umm, something that you probably should be testing for. Umm, I can’t say whether I’ve recently tested for arsenic in my well though I don’t really know much about, you know, how you remove it.*
Well water treatment	*The water up here, I understand, especially in the well water, must be tested by the city or town at a given period of time indicating to me that there’s some risk there otherwise you wouldn’t have to retest it as much as it is. And, um, I notice that most of the people up here have filtering systems on the faucet—osmosis type, chemical type, filtering type in their refrigerator, in their wells, even in their … I guess it’s city water here, where I’m living it’s a condominium community and I think we get our water from a source out of town*
Socioeconomic characteristics	*If you live in one the mobile home parks down here or one of the million dollar homes in a nicer area then you probably have a fancy filtration system that will stop you from getting it, so depending on your income* *So I think it’s not only less income but less educated people. People who don’t necessarily know the risks and aren’t going to, you know [referring to people buying lower quality food that might have more As in it]*
Geography (where you live)	*I know there’s natural levels of things that tend to come from granite rock I don’t know if arsenic is one of those compounds that tends to come from granite but, ahhh, that’s pretty much all I know about that.*
Historic land use	*no specific quote here but a good number of interviewees mentioned past apple orchards, a former farm with some contaminated sites, and one mentioned a tire pit.
Pesticides and contamination	*Sure, well, it’s used to kill, it used to be used to kill bugs in gardens so I’m assuming arsenic gets into the ground from that. Whether it dissipates from that, I don’t know. But I have a garden in the same place my father had a garden and he used arsenic.*
Regulations	*I would imagine the EPA probably takes some, I’m sure that they take some level of jurisdiction with regard to evaluating ground water for it. Quite honestly our neighborhood recently got town water established because there were, not so much arsenic, but they found other effluents in the ground as a result of leeching out of oil tanks and gasoline tanks.*
As in soil	*It could get into your crops/food if you are watering your food with contaminated water.*
As in surface water	*I don’t know if arsenic is prevalent in lake or river waters as much as it is from ground water but it is probably something they evaluate for.*
Apple crop practices	*Fruit just came to my mind. There is a lot of places that still use their insecticides and pesticides and preservatives. In that respect I would say that probably families with children who would be fed that kind of a diet would probably be more likely to have a lot more than they think of any kind of a poison.*
As in food and beverage	*But then I hear too, that rice has arsenic in it, so that would lead me to believe that it’s more of a … if rice has arsenic in it must be pulling it out of the soil. Might depend on the area but in that case I would that it would be more the topsoil, right? Or the water … rice is grown in a lot of water, right? So that seems more surface. I don’t know, I’m just speculating*
Age of exposure (fetal)	*If you were in an area where there was a higher concentration but then there are certain populations that might be more susceptible, like, you know, infants and children or people that are older or people that have compromised immune systems so there may be certain members of society that would be more susceptible to arsenic poisoning like little kids and lead exposures worse than an older person.*
Friends and neighbors	*One of the neighbors, I know, boils the water in order to get rid of the arsenic. They heard there was arsenic, they boil the water. I didn’t want to tell that probably concentrates the arsenic but that’s beside the point.*
Real estate	*Oh, well, when you buy a house you have the water tested. So I know that there are organizations that test drinking water and, I don’t know if the state does it, but I’m guessing that there’s probably something at UNH, that you can send water, I know that you can send soil and that’s through the COOP but I don’t know if they do water.*

## Supplementary Materials

A more detailed version of Figure 3 is available online at https://www.mdpi.com/1660-4601/16/18/3436/s1.
