# Supplementary material for: Communicating Arsenic’s Risks
Source: Int J Environ Res Public Health. 2019 Sep 16;16(18):3436. doi: 10.3390/ijerph16183436 (PMC6766008; doi:10.3390/ijerph16183436)
Supplement: Supplementary file 1 [file ijerph-16-03436-s001.pptx]

## Slide 1
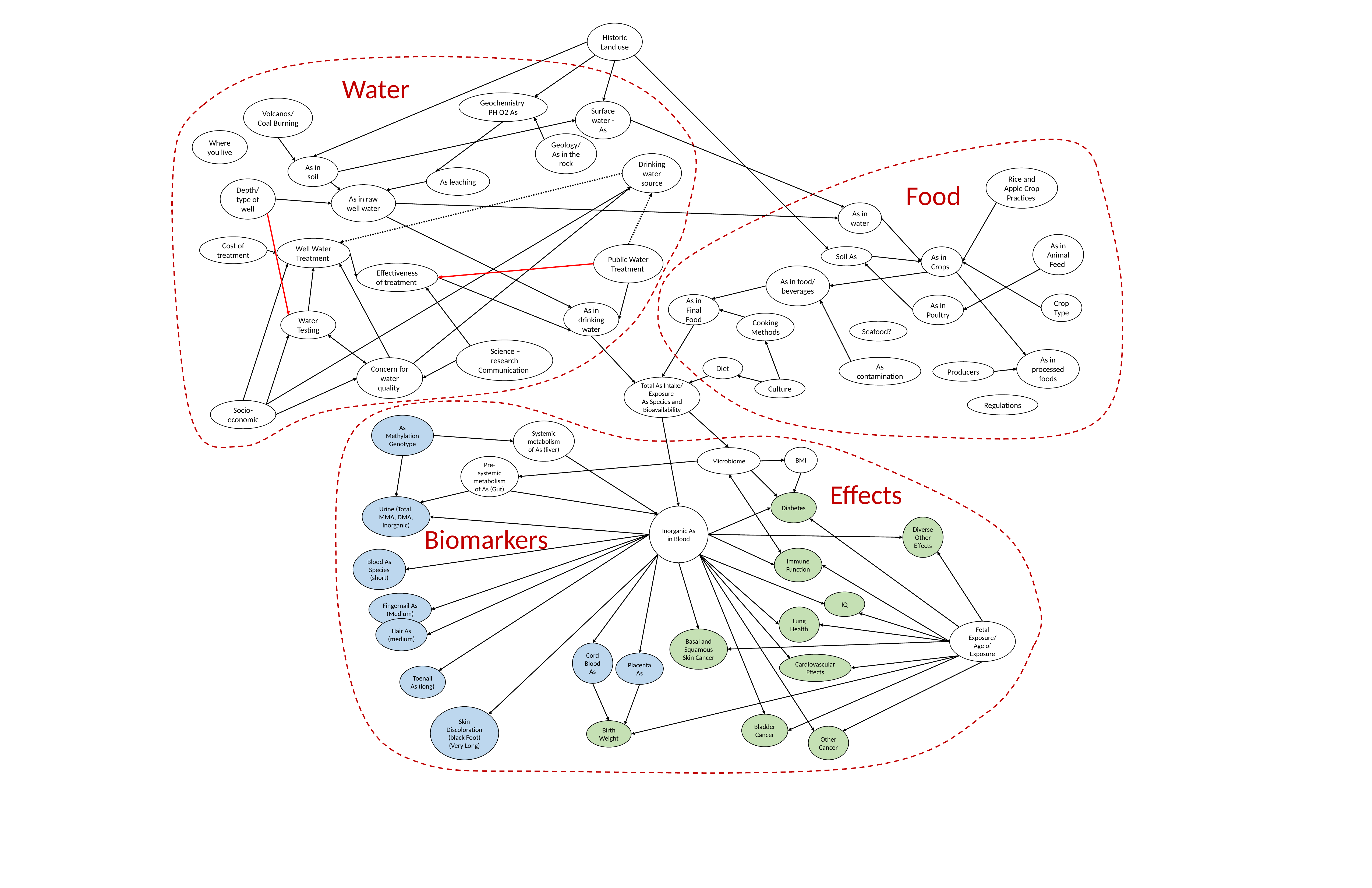

Historic Land use
Water
Geochemistry PH O2 As
Volcanos/ Coal Burning
Surface water - As
Where you live
Geology/ As in the rock
Drinking water source
As in soil
As leaching
Rice and Apple Crop Practices
Food
Depth/ type of well
As in raw well water
As in water
As in Animal Feed
Cost of treatment
Well Water Treatment
Public Water Treatment
Soil As
As in Crops
Effectiveness of treatment
As in food/ beverages
Crop Type
As in Final Food
As in Poultry
As in
drinking water
Water Testing
Cooking Methods
Seafood?
 Science – research Communication
As in processed foods
As contamination
Diet
Concern for water quality
Producers
Total As Intake/ Exposure
As Species and Bioavailability
Culture
Regulations
Socio-economic
As Methylation Genotype
Systemic metabolism of As (liver)
BMI
Microbiome
Pre-systemic metabolism of As (Gut)
Effects
Diabetes
Urine (Total, MMA, DMA, Inorganic)
Inorganic As in Blood
Diverse Other Effects
Biomarkers
Immune Function
Blood As Species (short)
IQ
Fingernail As (Medium)
Lung Health
Hair As (medium)
Fetal Exposure/ Age of Exposure
Basal and Squamous Skin Cancer
Cord Blood As
Placenta As
Cardiovascular
Effects
Toenail As (long)
Skin Discoloration (black Foot) (Very Long)
Bladder Cancer
Birth
Weight
Other
Cancer
